# Using Bandit Algorithms to Maximize SARS-CoV-2 Case-Finding: Evaluation and Feasibility Study

**DOI:** 10.2196/39754

**Published:** 2023-08-15

**Authors:** Michael F Rayo, Daria Faulkner, David Kline, Thomas Thornhill IV, Samuel Malloy, Dante Della Vella, Dane A Morey, Net Zhang, Gregg Gonsalves

**Affiliations:** 1 Department of Integrated Systems Engineering College of Engineering The Ohio State University Columbus, OH United States; 2 College of Public Health The Ohio State University Columbus, OH United States; 3 Department of Biostatistics and Data Sciences Wake Forest University School of Medicine Winston-Salem, NC United States; 4 Public Health Modeling Unit Department of the Epidemiology of Microbial Diseases Yale School of Public Health New Haven, CT United States; 5 Battelle Center for Science, Engineering, and Public Policy John Glenn College of Public Affairs The Ohio State University Columbus, OH United States

**Keywords:** active surveillance, bandit algorithms, infectious disease, community health, reinforcement learning, COVID-19, SARS-CoV-2

## Abstract

**Background:**

The Flexible Adaptive Algorithmic Surveillance Testing (FAAST) program represents an innovative approach for improving the detection of new cases of infectious disease; it is deployed here to screen and diagnose SARS-CoV-2. With the advent of treatment for COVID-19, finding individuals infected with SARS-CoV-2 is an urgent clinical and public health priority. While these kinds of Bayesian search algorithms are used widely in other settings (eg, to find downed aircraft, in submarine recovery, and to aid in oil exploration), this is the first time that Bayesian adaptive approaches have been used for active disease surveillance in the field.

**Objective:**

This study’s objective was to evaluate a Bayesian search algorithm to target hotspots of SARS-CoV-2 transmission in the community with the goal of detecting the most cases over time across multiple locations in Columbus, Ohio, from August to October 2021.

**Methods:**

The algorithm used to direct pop-up SARS-CoV-2 testing for this project is based on Thompson sampling, in which the aim is to maximize the average number of new cases of SARS-CoV-2 diagnosed among a set of testing locations based on sampling from prior probability distributions for each testing site. An academic-governmental partnership between Yale University, The Ohio State University, Wake Forest University, the Ohio Department of Health, the Ohio National Guard, and the Columbus Metropolitan Libraries conducted a study of bandit algorithms to maximize the detection of new cases of SARS-CoV-2 in this Ohio city in 2021. The initiative established pop-up COVID-19 testing sites at 13 Columbus locations, including library branches, recreational and community centers, movie theaters, homeless shelters, family services centers, and community event sites. Our team conducted between 0 and 56 tests at the 16 testing events, with an overall average of 25.3 tests conducted per event and a moving average that increased over time. Small incentives—including gift cards and take-home rapid antigen tests—were offered to those who approached the pop-up sites to encourage their participation.

**Results:**

Over time, as expected, the Bayesian search algorithm directed testing efforts to locations with higher yields of new diagnoses. Surprisingly, the use of the algorithm also maximized the identification of cases among minority residents of underserved communities, particularly African Americans, with the pool of participants overrepresenting these people relative to the demographic profile of the local zip code in which testing sites were located.

**Conclusions:**

This study demonstrated that a pop-up testing strategy using a bandit algorithm can be feasibly deployed in an urban setting during a pandemic. It is the first real-world use of these kinds of algorithms for disease surveillance and represents a key step in evaluating the effectiveness of their use in maximizing the detection of undiagnosed cases of SARS-CoV-2 and other infections, such as HIV.

## Introduction

### Background

Columbus, Ohio—like much of the world during the first 3 years of the pandemic—experienced multiple waves of infections in which the number of SARS-CoV-2 infections overwhelmed and outstripped the capacity of efforts to detect new cases [1,2]. In fact, estimates suggest that even at the height of the Omicron surge, a significant number of infections went undetected by case-based surveillance systems and the rise of at-home testing [3]. As the epidemic continues, particularly as public testing sites are scaled back and closed, the ability to quickly identify individuals with SARS-CoV-2 and link them to treatment has become difficult, risking patient and population health [4,5]. Given the integral role of active surveillance efforts in identifying cases, linking patients to care, and managing COVID-19, local public health departments require ways to maximize the resources they have at their disposal to continue to find new cases of disease in their jurisdictions even as resources for these efforts continue to shrink [4,6,7].

How to optimize resource allocation over time is a well-studied problem in sequential decision-making and reinforcement learning. The introduction of a spatial component to these kinds of dilemmas has been applied in a variety of settings, from military search and rescue to oil exploration [8]. We have previously described the use of one set of tools, bandit algorithms, to address these kinds of problems for detection of HIV and SARS-CoV-2 in the community [8-10]. Up until now, these methods have only been evaluated in computer simulations. The study described here represents the first-ever real-world implementation that investigates these tools for active infectious disease surveillance.

An academic-governmental partnership—the Flexible Adaptive Algorithmic Surveillance Testing (FAAST) program—between Yale University, Ohio State University (OSU), Wake Forest University (WFU), the Ohio Department of Health (ODH), the Ohio National Guard (ONG), and the Columbus Metropolitan Libraries (CML) conducted a study of bandit algorithms to maximize the detection of new cases in SARS-CoV-2 in Columbus in 2021.

This initiative began as the Delta variant of SARS-CoV-2 established itself in Ohio in the summer of 2021, ending a period of decreasing case numbers in the state. From mid-August to mid-September, the daily case count rose from over 2000 to over 9000 [11]. Around the same time, the ODH started distributing home rapid antigen tests (RATs) for COVID-19 to allow people to monitor their own health status. These tests were provided to community-based organizations in Ohio, including over 246 library locations, which received more than 53,000 rapid tests in late summer 2021 [12]. While the high demand meant that these libraries quickly ran out of tests to distribute, the community’s relationship and trust in libraries as a testing resource for COVID-19 enabled us to partner with the CML for this study [13]. The strong uptake of RATs is consistent with evidence that voluntary use of these tests is high in settings where they have been widely distributed [14], but it is important to note that decisions to opt in to population-level voluntary testing programs appear to be influenced by factors such as socioeconomic status rather than infection risk alone [15]. Thus, active surveillance has an important role in both prioritizing limited testing resources and in reaching individuals not seeking voluntary testing.

Working with our partners, we were able to offer an option to maximize the effectiveness of the scarce testing resources in Columbus by feeding our daily testing information into the bandit algorithm to target the next day’s testing efforts. Our “learn by doing” method adaptively targeted locations in the CML system, with tests supplied by ODH and performed by ONG with student and faculty support from OSU, WFU, and Yale.

As the COVID-19 pandemic continues, we will further evaluate the tools in Columbus and the state of Ohio and elsewhere as opportunities arise. Given the flexibility of these methods, we can tailor them to identify new cases more efficiently in underserved communities, which may be at higher risk of transmission and serious clinical disease either due to undervaccination and the prevalence of underlying conditions or due to specific kinds of workplaces and high-risk settings (eg, skilled nursing facilities). Importantly, the potential utility of these tools is not limited to SARS-CoV-2. Evaluation of these tools for HIV, hepatitis C virus, and other sexually transmitted diseases is also being considered.

### Goals of the Study

The purpose of this study was to evaluate the use of bandit algorithms to maximize the yield of testing for SARS-CoV-2 across multiple community sites in Columbus, Ohio, over a 3-month period from August to October 2021.

We sought to understand (1) if an algorithm-guided site selection would be operationally feasible for the branches of the CML system and ONG, since locations could shift from day to day, requiring staff to be ready and prepared for quick deployment and setup of testing efforts; (2) if shifting locales would present difficulties for uptake of testing services by the community, given the fact that the announcement of testing locations would only occur a few days in advance; and (3) how algorithm-guided site selection impacted the recruitment of minority residents of underserved communities in terms of number of tests conducted at each event among key demographic groups, particularly African-Americans, in Columbus.

In general, we were interested in a better understanding of the operational performance of the algorithm—as a formal analysis of the effectiveness of this algorithm-guided site selection approach to active disease surveillance would require a large cluster-randomized trial. Specifically, we chose 2 a priori indicators that the algorithm was homing in on hotspots for detecting new cases. Our goal was to sustain a testing positivity rate that was greater than (1) the estimated prevalence of the surrounding community and (2) the positivity rates of other types of testing events in the same zip codes. It is important to clarify that we were not attempting to estimate local prevalence of disease or positivity rates in this study nor making inferences about the effectiveness of the algorithm in practice but using these 2 metrics as a guide as to its basic performance as we assessed its initial feasibility in this research. The algorithm is a sequential decision-making tool; in this case, it was designed to guide resource allocation decisions for SARS-CoV-2 testing efforts.

## Methods

### Statistical Approach

The algorithm used to direct pop-up SARS-CoV-2 testing for this project has been described in detail elsewhere [8-10]. The algorithm is based on Thompson sampling, which uses a Bayesian updating process involving iteratively sampling from prior probability distributions of all potential testing sites—the set of all locations at which testing is being considered—to home in on those with the highest probability over the long run in finding new cases of SARS-CoV-2 [16,17]. Multimedia Appendix 1 describes Thompson samplng in more detail. This approach is not being used to estimate the local prevalence or test positivity at each site in the community but to maximize success in finding new cases over time. This algorithm is not a sampling strategy in which we seek to learn about an underlying population parameter (eg, prevalence) but simply a process by which we can direct testing efforts most efficiently. In fact, the reason this algorithm is not designed to estimate local prevalence is because this metric, while related to testing yield, may be confounded by other factors at work in a given location. That is, while testing yield is indeed a function of prevalence in part, prevalence does not provide a one-to-one proxy for the number of tests obtainable at a given location. For instance, a given site may have a lower prevalence than another but be a place in which more new diagnoses are garnered for other reasons (eg, higher health-seeking behavior, fewer options for testing in other settings); thus, this algorithm is designed to maximize testing yields alone.

Testing sites are determined a priori and serve as a fixed list of potential locations for deploying testing. Initial prior probability distributions for each site can be assigned to be noninformative (ie, uniform), reflecting a lack of information about where yield might be highest, or informative based on local knowledge of the pandemic, previous testing efforts, or social and economic characteristics of a given neighborhood. While informative priors can aid convergence on hotspots if the information is accurate, they can delay convergence if the information is incorrect, so one should use caution when specifying the initial prior distributions.

For this study, we chose to specify informative prior distributions for each site based primarily on results from previous feasibility testing events that we conducted at each site prior to use of the algorithm, with slight modifications to account for the current pandemic conditions. To estimate the baseline values for the parameters for our prior distributions for each potential testing site, we consulted with experts on the local epidemiological conditions and considered the prior testing event results, the reported rate of SARS-CoV-2 infections, and the vaccination rate in the neighborhoods surrounding each site. Taken together, this process yielded assumed β distributions for the positivity rate at each site where the α parameter represented the assumed number of positive tests and the β parameter the assumed number of negative tests. The assumed positivity was α / α + β and the total number of tests (ie, the sum of the α and β parameters). Details of the derived values for each site are shown in the Results section. While the true values of these parameters are unknown, we decided that given the short duration of our study period and the quality of the empirical and expert knowledge on these testing sites, estimating the values for these initial α and β parameters for each location was a reasonable choice.

**Figure 1:**
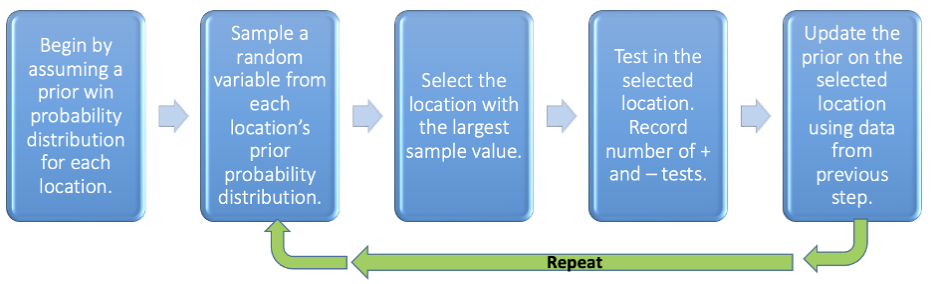
Steps in the implementation of the Thompson sampling algorithm.

Before assigning a location for a given day’s testing efforts, the algorithm randomly samples from the probability distributions of all the potential testing sites. Then, the site which has the largest realized value from these random draws is selected for the day’s testing deployment [18]. The data on the number of tests at that day’s site and the number of new diagnoses among them is then used to update the prior probability distribution for the site. The resulting posterior distribution after that day’s testing then becomes the updated prior distribution for the next day’s testing deployment. Before the next outing, a random draw from all the sites’ updated probability distributions happens again. This process is then repeated so the algorithm updates with each subsequent testing deployment. The entire procedure is described in Figure 1. The intuition here is that over time, using the knowledge accrued by each outing’s successes or failures (positive and negative tests), the algorithm learns the expected yields at each site and refines the probability distributions used for the random draws. Though all sites will have a nonzero chance of being chosen, the Bayesian updating processes here will be drawn toward sites where successes are occurring in previous rounds of testing. What is important is that other sites can still be chosen by chance and offer the opportunity to keep gathering information on the entire landscape of testing sites, but through the algorithm, we will prioritize the ones with the current greatest yields. If a low-priority site gets picked by random draw and turns out to have a high yield of positive tests, that site’s probability distribution now will shift its likelihood of being chosen in subsequent rounds.

**Figure 2:**
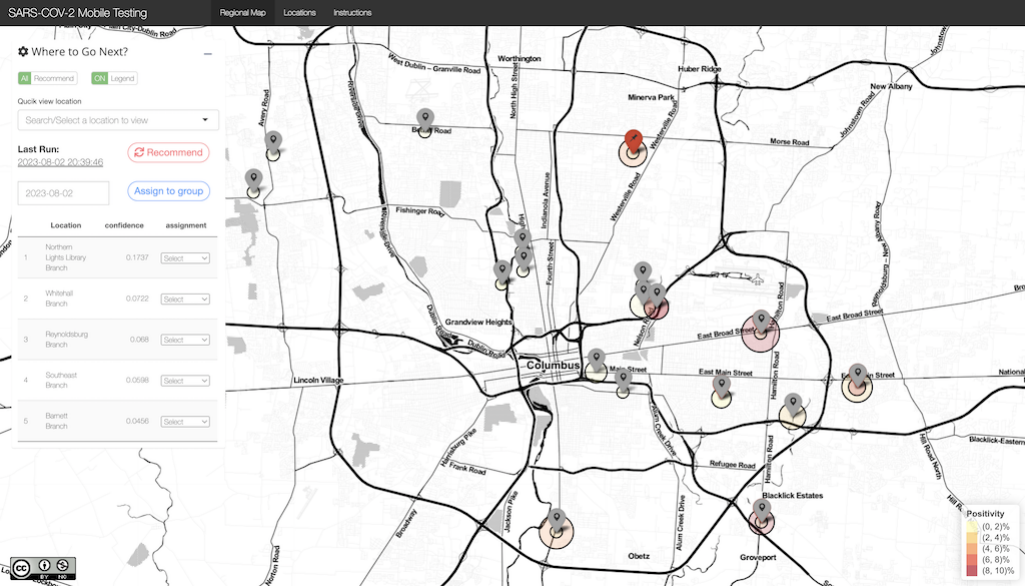
Regional map page of the web app for targeting SARS-CoV-2 testing with mobile units.

**Figure 3:**
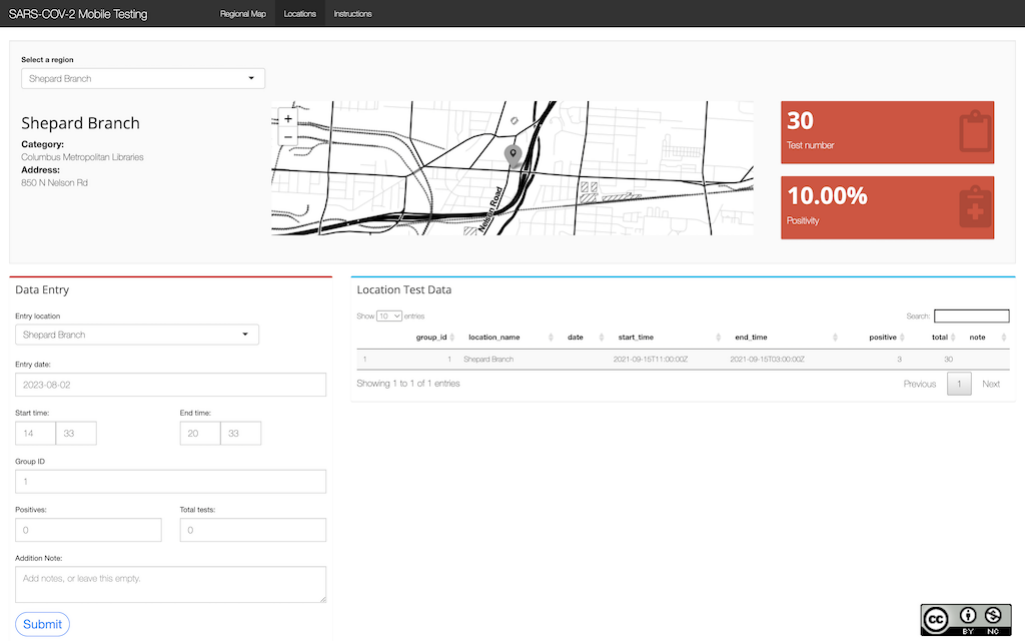
Locations page of the web app for targeting SARS-CoV-2 testing with mobile units.

Based on this algorithm, a web app was created for this project [19]. The underlying code is also available [20]. The app requires data input from each location where a testing event took place. Based on the information received, the tool then suggests testing locations likely to yield the most undetected positive cases per test for the next event. Views of the app’s regional and location pages are presented in Figures 2 and 3, respectively. Figure 2 shows a map of Columbus with pinned testing locations. After each testing event, data from the outing were entered into the algorithm in Figure 3 (eg, date and time, the total number of tests conducted, and number of positives). The app then integrates this data into the bandit algorithm, which delivers a new testing location for the next outing. Given conjugacy of the underlying Bayesian model, the computational cost of this process is minimal, as it only requires the ability to generate random draws from a beta distribution.

### Program Design and Implementation Overview

The logic model for program design and implementation is shown in Textbox 1. The logic model depicts the program events and expected outcomes through the integration of resources, the execution of activities, and the participation of communities and community organizations, resulting in measurable near-term and long-term outcomes. The inputs include program partners and the resources they provided to the project. The activities represent the main programmatic tasks accomplished with the participation of those tested in underserved communities in Columbus and with the assistance of local community-based organizations. The achievements of the program are denoted in short-term, measurable outcomes and benefits to participants and the larger community, as well as long-term, large-scale outcomes and benefits for the city and beyond.

The project team established partnerships with ODH, ONG, the CML system, and the Columbus Department of Neighborhoods (CDN). ODH supported the project by providing BinaxNOW RATs (Abbott), while ONG became a clinical partner who deployed their clinical staff to the sites to conduct testing. The neighborhood liaisons from CDN consulted the team regarding the initial location selection and promoted testing events in their designated areas of oversight. Finally, CML provided 7 of their branch locations as sites for pop-up testing and promoted the testing events through the CML network.

**Figure 4:**
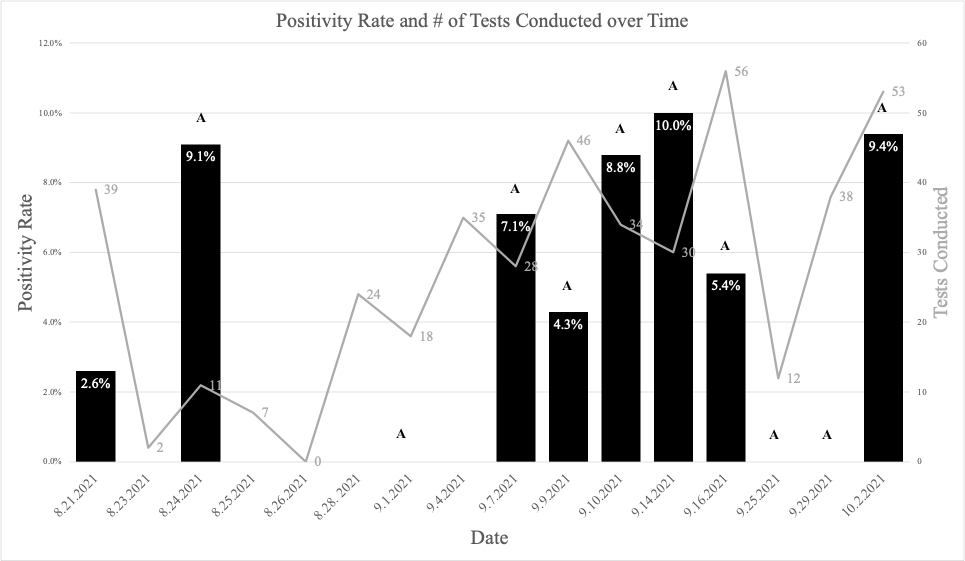
Positivity rate (black bars) and tests conducted (grey lines) for each testing session. The letter A is used to indicate testing at a library location. Starting on September 14, 2021, all testing locations were chosen by the algorithm.

The study lasted 3 months, from August to October 2021. The duration of each testing event was about 4 hours each day. We conducted a total of 16 testing events at 13 zip codes. Figure 4 shows dates of testing events, tests conducted, number of positive SARS-CoV-2 diagnoses, and the site positivity rate. Multimedia Appendix 2, Table S1 contains information about the sites and the zip codes where they were located.

**Logic model for the program and its implementation.**

**Inputs**

- Resources
  Research and statistical design
  Funding
  Staffing
  Mobile van
  Logistics
  Testing sites
  Rapid tests
  Personal protective equipment
  Clinical support
- Partners
  Ohio State University
  Yale University
  Wake Forest University
  Columbus Metropolitan Libraries
  Ohio Department of Health
  Ohio National Guard

**Processes**

- Activities
  Build the application
  Conduct pop-up COVID-19 testing events
  Distribute COVID-19 home test kits
  Disseminate information about COVID-19
- Participants
- Target population: underserved communities in Columbus
- Community organizations (eg libraries, parks, and recreation centers)

**Outcomes**

- Near-term
  Consistent detection of undiagnosed cases with the positivity rate being close to the state’s rate
  Increased community access to COVID-19 rapid home tests
  Increased community awareness about COVID-19 testing opportunities
- Long-term
  Convenient and effective testing in low-opportunity areas in Columbus, Ohio
  Prevention of transmission of COVID-19 in Columbus
  Use of bandit approach in other infectious disease surveillance efforts

The initial choice of testing sites was derived from a set of highly trafficked candidate locations based on raw cellphone data from the UberMedia COVID-19 recovery data set spatially joined with Loveland Landgrid parcel data to produce indices of contacts and unique contacts per parcel ranked by volume [21,22]. From this list, 30 of the most highly trafficked locations from the data set were selected for further analysis. A team of volunteers from OSU then visited each location to assess the viability of each site in terms of the availability of the venue (whether it was private or public, its accessibility, its hours of operation, and the availability of onsite parking). The final step of the selection process was a discussion of each site with the neighborhood liaisons from the CDN, who shared their insights about the proposed locations and the potential for attracting participants to come forward for testing at each site.

**Figure 5:**
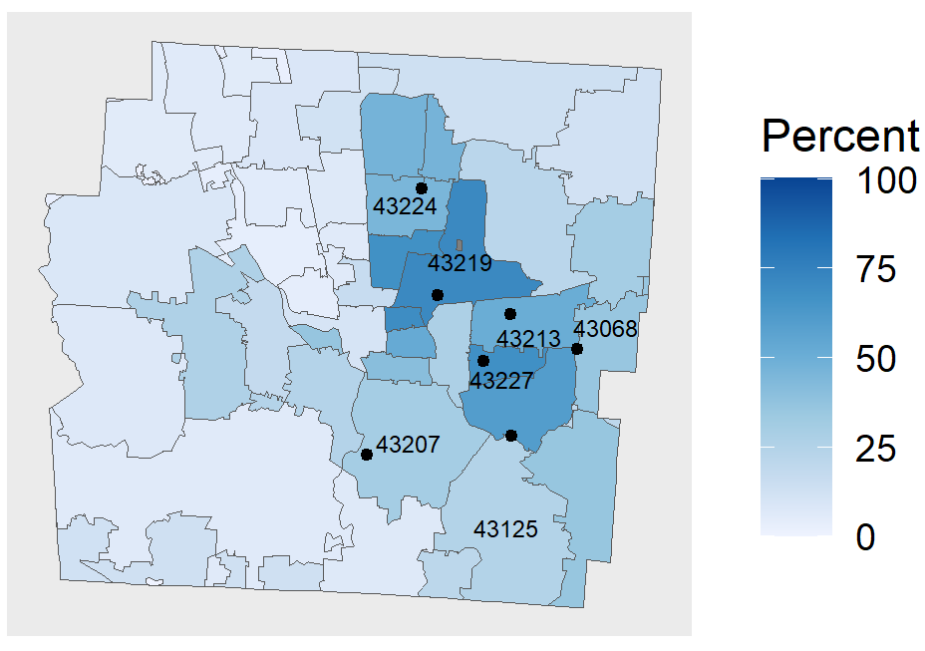
Location and zip codes of final testing sites with percentage of Black or African American residents.

Each testing event required the deployment of 2 volunteers from OSU and 2 ONG staff members. At the start of the study, events took place 2 to 3 times a week at community centers, movie theaters, parks, community events, shelters for the homeless, and libraries. These early weeks were dedicated to exploring the viability of the sites, constructing prior probability distributions for the algorithm for each site, and establishing an efficient operational workflow. In the last 2 weeks of the study, we deployed the bandit algorithm to choose sites for testing, restricting our sites to the branches of the CML, which turned out to be the most suitable sites for SARS-CoV-2 testing in the previous weeks. In these last weeks, the algorithm chose to send the team to sites 2, 4, 5, and 7, in that order, from September 16 to October 2, 2021 (site attributes can be found in Table 1). CML became an active partner in these efforts, promoting the testing events throughout their system with posters announcing the upcoming testing events distributed among the libraries in English, French, and Spanish. Figure 5 shows the location of the CML branches and underlying demographic information about these zip codes.

**Table 1:** Parameters for the assumed prior probability distributions for each testing site.

Site	Positivity	Tests, n	α	β
1	0.100	60	6	54
2	0.055	55	3.025	51.975
3	0.080	20	1.6	18.4
4	0.095	40	3.8	36.2
5	0.100	60	6	54
6	0.090	40	3.6	36.4
7	0.090	30	2.7	27.3

Testing efforts were focused on reaching underserved communities through strategic selection of potential sites; however, testing was open to everyone. To invite undocumented immigrants to be tested as well, participants were not required to present identification documents. Results of the tests were entered in SimpleReport—a free tool built by the US Centers for Disease Control and Prevention that makes it easy for COVID-19 testing sites to record results for rapid point-of-care tests and quickly report required data to public health departments [23].

### Ethical Considerations

The Office of Responsible Research Practices and the institutional review board at the Ohio State University deemed this project (2021E0496) exempt and not subject to regulations requiring institutional review board review and approval.

## Results

We set a baseline goal of testing approximately 20 people at each event through consultation with other institutions that have deployed mobile pop-up testing sites (eg, New York City Health and Hospitals Corporation, University of Cincinnati Medical Center). Of 16 testing events conducted, 10 testing events met the baseline goal of testing over 20 participants. The total number of people tested over the course of the study was 405 (the mean number of tests conducted at each event was 25.3), producing 20 positive tests. This gave us an average positivity rate of 4.9% across all sites, with the highest being 9.5%. The positivity rates observed at our testing sites were comparable to or greater than the officially documented positivity rates in those same areas in central Ohio on the days of our testing events [24-26]. In particular, the proportion of symptomatic positive tests in our study exceeded those obtained from other providers in the state, which prioritized symptomatic individuals for diagnosis [27]. The rolling 2-week average positivity rate for all tests conducted in our study ranged from 2.9% (3/105) to 6.5% (9/138); for asymptomatic individuals, positivity ranged from 0% to 5% (3/57); and for symptomatic individuals, the range was from 0% to 21.4% (3/14). In each week of testing, and in each rolling 2-week period, the positivity of symptomatic testing was greater than the daily rate range of the surrounding county. In the final 2 weeks, when the bandit algorithm was used to select testing event sites, the average positivity rate for symptomatic testing was 17.4 (4/23)%, which nearly doubled the highest recorded daily positivity rate in Franklin county at that time (Table 1) [24-26]. Details are shown in Table 2.

Underrepresented communities were reached at testing events at rates higher than or near their proportion of the population in the zip codes we tested in. The percentage of Black or African American people tested was larger than the corresponding percentage of African Americans in 3 of 5 zip codes in which our testing sites were located [28]. Details are shown in Table 3.

**Table 2:** Study positivity rates compared to tests conducted by other providers in Franklin County, Ohio.

		Positivity for rolling 2-week periods in 2021							
		Aug 12-25	Aug 19-Sept 1	Aug 26-Sept 8	Sept 2-15	Sept 9-22	Sept 16-29	Sept 23-Oct^a^	Total
**Flexible Adaptive Algorithmic Surveillance Testing clinic** **, n/N (%)**									
	All tests	3/65 (5)	5/96 (5.2)	4/99 (4)	8/146 (5.5)	9/138 (6.5)	3/105 (2.9)	6/106 (5.7)	22/408 (5.4)
	Asymptomatic individuals	3/57 (5)	4/82 (4.9)	1/85 (1.2)	3/116 (2.6)	3/105 (2.7)	0/78 (0)	2/83 (2.4)	9/330 (2.7)
	Symptomatic individuals	0/8 (0)	1/14 (7.1)	3/14 (21.4)	5/30 (16.7)	6/33 (18.2)	3/27 (11.1)	4/23 (17.4)	13/78 (16.7)
Franklin County daily rate^b^ (%), range		5.5-7.2	5.5-9	7-9.9	9.5-10.2	9.5-10.4	9.3-10.4	8.9-10	5.5-10.4

^a^Time period when bandit algorithm was used to select testing event sites.

^b^Absolute numbers were not available for county daily rate.

**Table 3:** Study representation of Black or African American people by zip code.

	Zip code, n/N (%)				
	43227	43125	43207	43068	43213
Black or African American participants in Flexible Adaptive Algorithmic Surveillance Testing clinics	18/24 (75)	17/31 (55)	8/31 (26)	13/29 (45)	13/28 (46)
Black or African American population of Franklin county (US Census; 2021 American Community Survey 5-year data)	16,740/24,779 (67.6)	3429/13,756 (24.9)	13,936/46,308 (30.3)	20,658/58,426 (35.4)	18,205/35,954 (50.6)

Throughout the study, 55% (156/284) of the population tested identified themselves as African American, and 36% (103/284) self-identified as White. Almost half of the population tested (105/227, 46%) stated their level of education was high school or below, and 47% (106/225) stated they worked full-time. Data about vaccination status against COVID-19 was collected as well: 56% (126/224) were vaccinated, compared to the vaccination rate in Franklin County of 54% [29].

Our survey results indicate that 27% (41/153) of those who attended our testing events found out about the testing event from others, 22% (34/153) by communicating with the library, and 29% (45/153) by walking or driving by.

## Discussion

### Principal Findings

This study demonstrated that a pop-up testing strategy using a bandit algorithm can be feasibly deployed in an urban setting during a pandemic. Although this was a limited roll-out of the strategy, positivity rates in this feasibility study were comparable or greater to those obtained by other testing initiatives primarily targeted at symptomatic individuals at the same time in these same areas of central Ohio on the days of our testing events, even though the bulk of those tested at our sites were asymptomatic, providing encouraging, though only preliminary, evidence that this bandit algorithm may be useful in improving case detection efforts [24-26]. It is the first real-world use of these kinds of algorithms for disease surveillance and represents the first step in evaluating the effectiveness of their use in maximizing the detection of undiagnosed cases of SARS-CoV-2 and other infections, such as HIV. In addition, the study showed that a simple, scaled-down testing approach with a total budget of under US $10,000 could carry out a program that could test up to 60 people in a single 4-hour event during its 3-month run. This shows that a flexible, adaptive approach to SARS-CoV-2 surveillance can be run efficiently with support for basic supplies (eg, tents, chairs, tables), and partnerships with organizations who could supply the tests (ODH) and carry them out (ONG) [30].

The program’s ability to reach underserved communities is especially notable for several reasons. The quick-pivot nature of the program required that we give communities very little notice of upcoming events, potentially taxing their ability to hear about and be able to travel to locations to participate. Accessibility and transportation are barriers to health services in some of these communities [31,32]. In addition, there have been nationwide and state-wide trends of COVID-19 resources being allocated inequitably toward wealthier, predominantly White communities [33]. This is particularly troubling as both general determinants of health as well as the specific impact of COVID-19 make communities of color and other underserved communities more vulnerable to COVID-19 infection and the morbidity and mortality associated with the disease [34,35].

Libraries are important resources for communities in the context of population health [36,37]. A novel discovery during the study was the importance of the CML system in our active disease surveillance efforts. We learned that the CML is known and trusted by the communities of the city as a source of information and a site for social services. Subsequent studies in Columbus will now be done in partnership with the CML, as it excels at community outreach, has a network of facilities strategically placed in targeted neighborhoods, communicates well within its network, and is eager to work with public health partners. Our testing partner, ONG, was suitable for this study, but because of other commitments, we could not deploy the algorithm on less than 3 days’ notice with them, and we are seeking out partners (a local federally-qualified health center) for the next phase of this work that have greater flexibility and can be available on short notice. While a major strength of our approach is its ability to deploy testing teams to locations based on emerging data in real time, it requires an operational nimbleness from partners, which may not be possible for some.

This study uses a novel approach to implementation science in that it addresses feasibility and acceptability of a new strategy for active surveillance of infectious diseases as a precursor to additional evaluation of the effectiveness of these algorithms in the field. Randomized controlled trials (RCTs) are expensive to mount and time consuming for both researchers and participants—this kind of preliminary research on implementation challenges is critical to understand the contextual factors that might present difficulties for the conduct of RCTs or real-world use of the intervention under study [38].

### Limitations

This study has a number of limitations. First, it was conducted over a comparatively short period of time at a specific point of increasing concern in the pandemic. As such, the engagement of the community and the effectiveness of the algorithm must be set within that context. In addition, this study was only conducted in one midsized Midwestern city and may not be generalizable to other settings.

### Conclusions

Through the implementation of a bandit algorithm in this study, we demonstrated the feasibility of such approaches to guide community testing for SARS-CoV-2. In addition, we established an efficient workflow and operational plan that can be extended to other organizations conducting mobile testing. In particular, we were pleased that the program appears to be well-suited to reach historically underserved communities in Columbus. As the COVID-19 pandemic persists in the United States, using our approach not only for active surveillance but as a core component of “test-to-treat” or “test-to-vaccinate” efforts targeted at the communities most at risk of the disease should be considered as well [39,40]. Finally, this algorithmic approach is pathogen-agnostic—it can be used for other infectious disease efforts. It was initially developed for maximizing the yield of undiagnosed HIV infections but could be used similarly for other infections to maximize case detection in the community (eg, for sexually transmitted diseases) or as an adjunct to other efforts, beginning with case identification (eg, test-to-treat for SARS-COV-2, HIV, hepatitis C virus, and sexually transmitted infections). The important next step is to evaluate the effectiveness of this algorithm in its ability to maximize the number of cases of disease detected in comparison to standard operating procedures for such deployment by public health practitioners. As an interim step, we have established a new partnership with a public health department in one of the largest US cities and will be evaluating the performance of Thompson sampling using historical data provided by it to compare how yields of testing using this algorithm would have differed from the actual number of positive tests collected for SARS-CoV-2 over the past 3 years with their fleet of mobile health vans. Depending on the outcome of this analysis, we will pursue funding for a prospective study of our approach in the context of a cluster randomized trial.

## Appendix

Multimedia Appendix 1 Thompson sampling algorithm.

Multimedia Appendix 2 Table S1. Location data from the conducted testing events.

## Abbreviations

CDN: Columbus Department of Neighborhoods
CML: Columbus Metropolitan Libraries
FAAST: Flexible Adaptive Algorithmic Surveillance Testing
ODH: Ohio Department of Health
ONG: Ohio National Guard
OSU: The Ohio State University
RAT: rapid antigen test
RCT: randomized controlled trial
WFU: Wake Forest University

## Data Availability

All data generated or analyzed during this study are included in this published article and its supplementary information files.
