# Supplementary material for: Using Bandit Algorithms to Maximize SARS-CoV-2 Case-Finding: Evaluation and Feasibility Study
Source: JMIR Public Health Surveill. 2023 Aug 15;9:e39754. doi: 10.2196/39754 (PMC10430782; doi:10.2196/39754)
Supplement: Multimedia Appendix 1 [file publichealth_v9i1e39754_app1.docx]

| **Algorithm:** Thompson sampling Strategy |
| --- |
| For each location *i = 1, 2, 3* set *Xi(0)=0, Yi(0)=0*.  **for each** *t = 0, 1, 2…tmax,* **do**  For each location *i = 1, 2, 3,* sample *θi(t)* from the Beta *(αi + Xi(t), βi + Yi(t))* distribution.  Select location *j=argmaxi θi(t).*  Perform *m* Bernoulli trials in location *j* with success probability *Pj(t)* and observe *xj* successes and (*m-xj*) failures.  Let *Xj(t + 1) = Xj(t) + xj* and *Yj(t + 1) =Yj(t) + (m-xj).*  For all locations *i ≠ j,* let *Xi(t + 1) = Xi(t)* and *Yi(t + 1) =Yi(t).*  **end** |
