# Supplementary material for: Using Bandit Algorithms to Maximize SARS-CoV-2 Case-Finding: Evaluation and Feasibility Study
Source: JMIR Public Health Surveill. 2023 Aug 15;9:e39754. doi: 10.2196/39754 (PMC10430782; doi:10.2196/39754)
Supplement: Multimedia Appendix 2 [file publichealth_v9i1e39754_app2.docx]

Supplemental Table: Locations data from the conducted testing events.

| ZIP Code | Location | Date | Tests Conducted | Positive Number | Positivity Rate |
| --- | --- | --- | --- | --- | --- |
| 43227 | Far East Community | 8.21.2021 | 39 | 1 | 2.6% |
| 43201 | Tuttle Park | 8.23.2021 | 2 | 0 | 0 |
| 43068 | CML Reynoldsburg | 8.24.2021 | 11 | 1 | 9.1% |
| 43212 | Lennox Movie | 8.25.2021 | 7 | 0 | 0 |
| 43220 | North Creek Park | 8.26.2021 | 0 | 0 | 0 |
| 43205 | Friends of the Homeless | 8.28.2021 | 24 | 0 | 0 |
| 43213 | CML Whitehall | 9.1.2021 | 18 | 0 | 0 |
| 43219 | YMCA | 9.4.2021 | 35 | 0 | 0 |
| 43224 | CML Northern Lights | 9.7.2021 | 28 | 2 | 7.1% |
| 43227 | CML Barnett | 9.9.2021 | 46 | 2 | 4.3% |
| 43125 | CML Southeast | 9.10.2021 | 34 | 3 | 8.8% |
| 43219 | CML Shepard | 9.14.2021 | 30 | 3 | 10% |
| 43207 | CML South High | 9.16.2021 | 56 | 3 | 5.4% |
| 43224 | CML Northern Lights | 9.25.2021 | 12 | 0 | 0 |
| 43068 | CML Reynoldsburg | 9.29.2021 | 38 | 0 | 0 |
| 43213 | CML Whitehall | 10.2.2021 | 53 | 5 | 9.4% |
